# A case report on atypical spindle cell lipomatous tumor: A rare entity

**DOI:** 10.1016/j.amsu.2022.104205

**Published:** 2022-07-16

**Authors:** Himal Bikram Bhattarai, Sangit Chhantyal, Krishna Dahal, Sangam Shah, Saroj Kumar Yadav, Binita Kumari Yadav, Ayusha Subedi, Santosh Baniya, Prabesh Bikram Singh, Anshu Priya

**Affiliations:** a Gandaki Medical College and Teaching Hospital, Pokhara, Nepal; b Tribhuvan University, Institute of Medicine, Maharajgunj, 44600, Nepal; c Department of General Surgery, Koshi Hospital, Biratnagar, Nepal; d Greencross Hospital, Biratnagar, Nepal; e Manmohan Memorial and Community Hospital, Jhapa, Nepal; f Janaki Medical College, Janakpur, Nepal

**Keywords:** Spindle, Lipomatous, Tumor

## Abstract

**Introduction:**

Atypical spindle cell lipomatous tumor (ASPLT), a separate entity for the group of benign/low grade adipocytic neoplasm that is characterized by adipocyte adequacy and the presence of lipoblast-like cells and spindle cells at varying degrees.

**Case presentation:**

Here, we report a rare case of 60 years old male with atypical spindle cell lipomatous tumor in the anterior abdominal wall.

**Discussion:**

Histopathology is the gold standard for establishing the diagnosis and grade of soft tissue tumor and consistent radiology-pathology correlation is essential to avoid any diagnostic pitfalls [1]. Ultrasound is preferred as an initial investigation for superficially located lesions.

**Conclusion:**

ASPLT show a wide variety of microscopic features, and differential diagnosis is important and difficult. Recognition of morphologic clues and immunohistochemistry/molecular tests to confirm the diagnosis.

## Highlights

• Histopathology is the gold standard for establishing the diagnosis and grade of soft tissue tumor.
• Ultrasound is preferred as an initial investigation for superficially located lesions.
• Atypical spindle cell lipomatous tumor (ASPLT), a separate entity for the group of benign/low grade adipocytic neoplasm.

## 1 Introduction

In 2020, World Health Organization (WHO) Classification of Soft Tissue and Bone Tumors, described an atypical spindle cell lipomatous tumor (ASPLT), a separate entity for the group of benign/low grade adipocytic neoplasm that is characterized by adipocyte adequacy and the presence of lipoblast-like cells and spindle cells at varying degrees [[1], [2], [3]]. This entity can affect both sexes, with a slight male predominance, including mostly middle-aged adults with a peak in the sixth decade [4]. The most common locations are the hand, foot, thigh, followed by the shoulder, forearm, lower leg, with head and neck area, genital area, trunk, and back are less-common locations [5]. Rarely cases are reported in the larynx, trachea, mediastinum, retroperitoneum, and appendix [5]. Here, we report a rare case of atypical spindle cell lipomatous tumor in the anterior abdominal wall. This case has been reported as per SCARE 2020 guidelines [6].

## 2 Case presentation

A 60-year-old male presented to the clinic via ambulance complaining of a gradually increasing lump on the left side of the anterior abdominal wall for the last 3 years. Initially it was painless. However, for the last 6 month he was having a dull ache constantly with occasional exacerbations in between. He had a small lump at the same site 5 years back for which he had undergone excision. The patient did not have a biopsy at that time. On examination 10 × 10 cm non tender lump with firm to hard consistency was found on the left side of the anterior abdominal wall in between costal margin and iliac crest. The mass was found to be fixed to the abdominal wall muscles. The overlying skin showed scars of previous surgery. There were multiple small hard nodules in the vicinity of the main lump. Rest of the physical examinations were unremarkable.

**Fig. 1:**
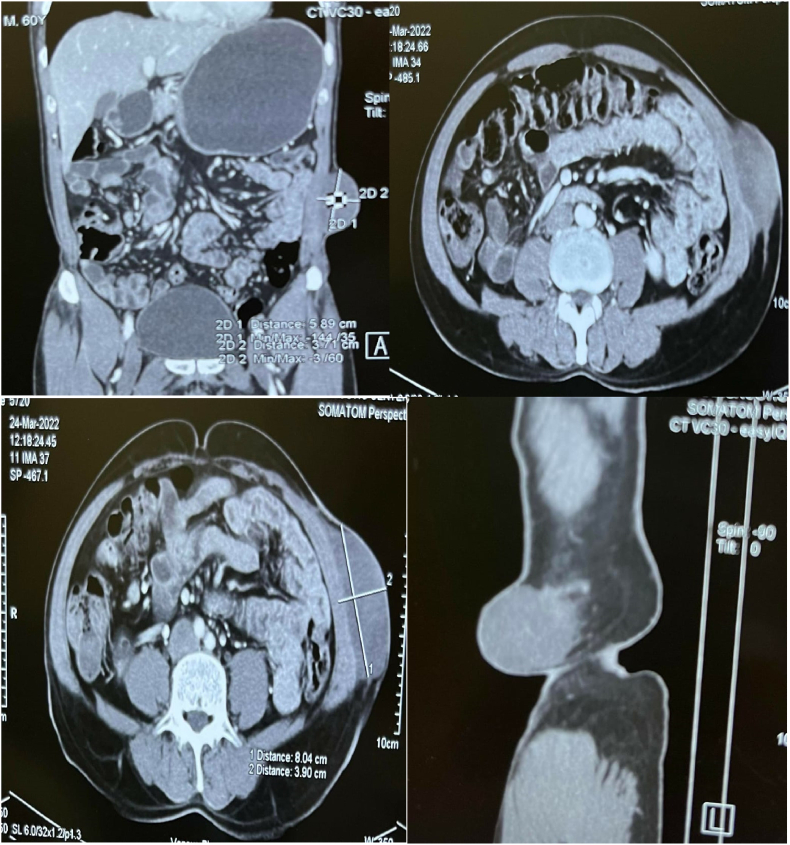
MPCT scan of abdomen (a) non contrast (b) contrast enhanced (c) Sagittarius (d) coronal view showing features of desmoid tumor.

Multi-phase computed tomography (MPCT) scan of abdomen was done, and it showed features suggestive of desmoid tumor (Fig. 1). In addition, this patient had concurrent bilateral renal cortical cysts, umbilical hernia, and degenerative changes of lumbosacral spine as identified by MPCT scan. He was taken for surgery and wide local excision with 2 cm margin away from tumor border was conducted under general anesthesia. The mass was found to be arising from external and internal oblique muscles and aponeurosis which too were excised. The satellite nodules were also exercised. The defect was repaired with 20 × 20 cm prolene mesh and a surgical drainage was kept. Post operative care of the patient was done, and he was in good condition during follow up.

**Fig. 2:**
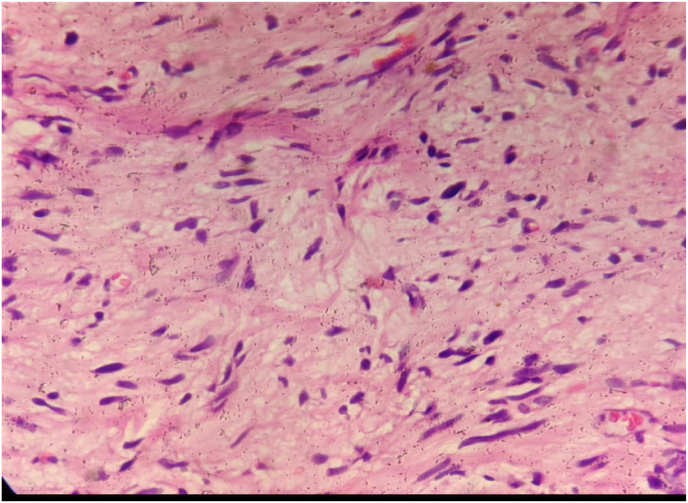
Histopathological examination showing the features of atypical spindle cell lipomatous tumor.

Histopathological examination of the tissue samples obtained from surgery showed well circumscribed mass consisting of spindle cells, adipocytes, and ropey collagen bundles in underlying fibromyxoid stroma. Individual spindle cells were bland, arranged randomly, and in short fascicles. Nuclei were uniform and elongated with bipolar eosinophilic cytoplasmic processes. Many hyperchromatic bizarre looking stromal cells, mature adipocytes and skeletal muscle bundles were seen (Fig. 2). Atypical cells, mitotic figures and necrosis are however absent in the entire section examined. These features were consistent with atypical spindle cell/pleomorphic lipomatous tumors. On follow-up after 2 weeks of surgery he was recovering well and upon two subsequent follow-up he had no complications.

## 3 Discussion

In 2010, Mentzel T et al. proposed the term “atypical spindle cell lipoma” for the first time, suggesting that these neoplasms most likely represent an independent entity which is closely related to spindle cell lipoma rather than to a morphologic variant of atypical lipomatous tumor/well-differentiated liposarcoma [7]. In 2020, WHO described an ASPLT as a new entity for the group of benign/low grade adipocytic neoplasm [1]. It has slight male predominance mostly middle-aged adults with a peak in the sixth decade [4]. It most commonly occurs in extremities with slightly more frequently in subcutis than in deep (subfascial) soft tissues [5].

Clinically ASPLT manifests a persisting or growing mass [2]. Complete surgical excision is the appropriate treatment for ASPLT. Incomplete surgical excision may cause local recurrence in 10–15% of the cases [4]. Our patient had recurrence of lump 5 year later at the same time however, no biopsy was done for the previous lump. The lump might have recurred now because he had incomplete excision earlier. Our patient had no metastasis, further supporting benign nature of ASPLT.

Histopathology is the gold standard for establishing the diagnosis and grade of soft tissue tumor and consistent radiology-pathology correlation is essential to avoid any diagnostic pitfalls [1]. Ultrasound is preferred as an initial investigation for superficially located lesions and magnetic resonance imaging (MRI) is the imaging gold standard and modality of choice for the evaluation of soft tissue tumors [1]. In our case, we chose to perform an excisional biopsy for easy access and since our main suspicion was a benign tumor.

The morphologic differential diagnosis of atypical spindle cell lipomatous tumor is broad, and includes spindle cell lipoma, diffuse neurofibroma, myofibroblastoma, Cellular angiofibroma, fat forming solitary fibrous tumor, Pleomorphic liposarcoma, atypical lipomatous tumors/well-differentiated liposarcoma and Dedifferentiated liposarcoma (ALT/WDLPS) [2,5].

Classic spindle cell lipoma is also a benign adipocytic tumor which in contrast with ASPLT arise on back of neck and posterior shoulder and lacks pleomorphic lipoblasts, atypical spindle cells, and “bizarre” pleomorphic stromal/multinucleated cells [5,8]. Diffuse neurofibroma, on the other hand, is a proliferation of S100 protein and CD34-positive spindle cells with wavy or buckled nuclei, often within dermis and subcutaneous tissue of young patients which can also be excluded from ASPLT as adipocyte differentiation is very rare in it [2]. Similarly, detection of hyperplastic nerve bundles and Meissnerian corpuscles further aid in identifying diffuse neurofibroma [2].

Myofibroblastoma of soft tissue is a benign neoplasm, occurring most often in the inguinal/groin areas [8] and Cellular angiofibroma is a benign, fibroblastic and vascular-rich neoplasm, arising in the superficial soft tissues of the vulvovaginal and the inguinoscrotal or para-testicular areas [9]. However, both these two entities can be differentiated from ASPLT as both show less-prominent fat component and CAF showing prominent hyalinized vessels [5]. Distinction from fat-forming solitary fibrous tumor (SFT) can be done as it occurs in deep soft tissues, shows variably prominent adipocytic component and nuclear expression of STAT6 detection by immunohistochemistry in virtually all SFTs [2].

In general, PLSs can be differentiated from ASPLTs by a higher degree of pleomorphism, high mitotic activity, and tumor necrosis [5]. Most patients of PLSs report a rapidly growing painless mass (median: 3–6 months) [4] in contrast to our case with history of 3 years. ALT/WDLPS is a locally aggressive adipocytic neoplasm distinguished from ASPLT by potential to dedifferentiate [1]. Dedifferentiated liposarcoma is characterized by abrupt transition from ALT/WDLPS to non-lipogenic sarcoma of variable histologic grade with a metastatic potential [9]. Both of these occurs most frequently in the retroperitoneum [9]. Amplification of MDM2 and/or CDK4 by FISH is important to distinguish these two entities from ASPLT [10,11].

## 4 Conclusion

Our study presents a rare case of a ASPLT in left anterior abdominal wall treated with complete excision. ASPLT show a wide variety of microscopic features, and differential diagnosis is important and difficult. Recognition of morphologic clues and immunohistochemistry/molecular tests to confirm the diagnosis. Although it is a benign adipocytic tumor, it carries a considerable risk of local recurrence if not completely excised.

## Ethical approval

None.

## Sources of funding

None.

## Author contribution

SS, SC, KD, and HBB wrote the original manuscript, reviewed, and edited the original manuscript. SKY, BKY, AS, SB, PBS, and AP reviewed and edited the original manuscript.

## Registration of research studies

1. Name of the registry: None
2. Unique Identifying number or registration ID: None
3. Hyperlink to your specific registration (must be publicly accessible and will be checked):

## Provenance and peer review

Not commissioned, externally peer-reviewed.

## Consent

Written informed consent was obtained from the patient for publication of this case report and accompanying images. A copy of the written consent is available for review by the Editor-in-Chief of this journal on request.

## Guarantor

Dr. Himal Bikram Bhattarai.

## Declaration of competing interest

None.
